# Evaluation of electronic recruitment efforts of primary care providers as research subjects during the COVID-19 pandemic

**DOI:** 10.1186/s12875-022-01705-y

**Published:** 2022-04-28

**Authors:** Olena Mazurenko, Lindsey Sanner, Nate C. Apathy, Burke W. Mamlin, Nir Menachemi, Meredith C. B. Adams, Robert W. Hurley, Saura Fortin Erazo, Christopher A. Harle

**Affiliations:** 1 Department of Health Policy and Management, Richard M. Fairbanks School of Public Health, Indiana University, 1050 Wishard Blvd, Ste 6140, Indianapolis, IN 46202 USA; 2 Perelman School of Medicine, University of Pennsylvania, Philadelphia, PA USA; 3 Regenstrief Institute, Inc., Indianapolis, IN USA; 4 Department of Medicine, Indiana University School of Medicine, Indianapolis, IN USA; 5 Department of Anesthesiology, Wake Forest University School of Medicine, Winston Salem, NC USA; 6 Department of Public Health Sciences, Wake Forest University School of Medicine, Winston Salem, NC USA; 7 Department of Neurobiology and Anatomy, Wake Forest University School of Medicine, Winston Salem, NC USA; 8 Eskenazi Health Centers, Eskenazi Health, Indianapolis, IN USA; 9 Department of Health Outcomes and Biomedical Informatics, College of Medicine, University of Florida, Gainesville, FL USA; 10 University of Florida Health, Jacksonville, FL USA

**Keywords:** Primary care provider, Recruitment, Randomized clinical trial, Clinical decision support

## Abstract

**Background:**

Recruiting healthcare providers as research subjects often rely on in-person recruitment strategies. Little is known about recruiting provider participants via electronic recruitment methods. In this study, conducted during the COVID-19 pandemic, we describe and evaluate a primarily electronic approach to recruiting primary care providers (PCPs) as subjects in a pragmatic randomized controlled trial (RCT) of a decision support intervention.

**Methods:**

We adapted an existing framework for healthcare provider research recruitment, employing an electronic consent form and a mix of brief synchronous video presentations, email, and phone calls to recruit PCPs into the RCT. To evaluate the success of each electronic strategy, we estimated the number of consented PCPs associated with each strategy, the number of days to recruit each PCP and recruitment costs.

**Results:**

We recruited 45 of 63 eligible PCPs practicing at ten primary care clinic locations over 55 days. On average, it took 17 business days to recruit a PCP (range 0–48) and required three attempts (range 1–7). Email communication from the clinic leaders led to the most successful recruitments, followed by brief synchronous video presentations at regularly scheduled clinic meetings. We spent approximately $89 per recruited PCP. We faced challenges of low email responsiveness and limited opportunities to forge relationships.

**Conclusion:**

PCPs can be efficiently recruited at low costs as research subjects using primarily electronic communications, even during a time of high workload and stress. Electronic peer leader outreach and synchronous video presentations may be particularly useful recruitment strategies.

**Trial registration:**

ClinicalTrials.gov, NCT04295135. Registered 04 March 2020.

## Background

Recruiting individual healthcare providers, such as physicians, nurse practitioners, and other medical professionals, as *subjects* in research studies is often a challenging task [1]. Healthcare providers report numerous barriers to participation, including time constraints, low interest in research topics, concerns about relationships with patients, loss of professional autonomy, and reluctance to modify existing clinical workflows [2–5]. Furthermore, researchers often face additional barriers to recruiting healthcare providers, including difficulty gaining permission to enter healthcare facilities, obtaining accurate eligibility and contact information, persuading eligible providers to participate, and scheduling data collection [6]. Consequently, many studies using healthcare providers as subjects, even randomized controlled trials (RCTs) taking place in academic medical centers, fail to achieve recruitment targets, leading to extensions of timelines, reductions in statistical power, or wasted resources [7–9].

Current best practices for recruiting healthcare providers include using clinical champions to contact eligible providers and establishing relationships with clinic personnel [5, 10, 11]. These best practices resulted in development of the 7R framework that provides guidance on recruitment strategies pertaining to: relationships, reputation, requirements, rewards, reciprocity, resolution, and respect [11]. The 7R framework has been effective in recruiting healthcare providers and medical groups [5, 11], but several strategies rely on in-person interactions between the research team and eligible providers. To date, there is little evidence on how best to implement the 7R framework via electronic methods. Even less information about healthcare provider recruitment is available during times of particularly high workload and stress. The COVID-19 pandemic resulted in significant increases in providers’ workload, stress, and burnout, which may have further hindered their participation in research [12, 13]. Thus, researchers trying to recruit healthcare providers as subjects likely experienced additional logistical barriers due to healthcare facility closures, visitor restrictions, and other physical distancing policies [14]. In addition, existing literature minimally quantifies the effect of electronic recruitment strategies on healthcare providers recruitment success and costs of recruitment [2, 15, 16].

The purpose of the current study is to describe and evaluate the implementation of a primarily electronic provider recruitment strategy that was based on the 7R framework. In the fall of 2020, we recruited primary care providers (PCPs) for a pragmatic RCT of an electronic health record (EHR)-based decision support intervention. Due to COVID-19 restrictions, we modified our recruitment approaches to use exclusively electronic strategies. We report detailed metrics capturing the electronic strategies’ effectiveness and a cost estimate of our recruitment approach. We also describe challenges faced when adopting the 7R framework for predominantly electronic provider recruitment. Our findings are relevant for future researchers aiming to recruit providers as research subjects by estimating the time and resources required, especially during times of high workload and stress.

## Methods

### RCT overview

The goal of the RCT was to assess a clinician-facing electronic health record (EHR)-based decision support tool called The Chronic Pain OneSheet (i.e., OneSheet) [17]. OneSheet is a patient-level dashboard that aggregates information relevant to the CDC Guideline for Prescribing Opioids for Chronic Pain into a single view [18]. OneSheet was designed to help PCPs quickly access the clinical information needed to take evidence-based action. OneSheet allows PCPs to collect and review patients’ chronic pain history, treatment plan, treatment-related risks, outcomes, and goals more efficiently. The study’s primary outcome, constructed using EHR data, is the proportion of patients with chronic pain for whom a PCP conducted guideline-recommended goal setting, pain and function assessment, and opioid-related risk assessment. The study received institutional board approval at Indiana University and is registered with ClinicalTrials.gov (NCT04295135).

### Setting & eligibility criteria

Eligible PCPs included physicians, nurse practitioners, and physician assistants from general internal medicine or family medicine practices that provide primary care services to adult patients with chronic pain. PCPs were recruited from two separate academic health center sites across two states, including one of the largest safety-net health systems in the Midwest. Due to the pandemic, our recruitment of PCPs at the Midwest site was conducted electronically and is thus the focus of this study. The Midwest site is part of a health system that includes ten federally qualified community health centers that provide primary care services to about 85,000 patients per year, with about 290,000 visits in 2020 [19].

### Original recruitment strategy

The 7R framework guided our original pre-pandemic recruitment strategy [5, 11]. Briefly, the 7R framework consists of seven strategies: 1) *Relationship*: recruiters need to be known for their involvement in the local medical community and for doing practical research, 2) *Reputation*: recruiters need to be known for doing research and participants need to believe that the relationship and information will not be abused, 3) *Requirements*: resource demands for participants in study-related activities need to be minimized, 4) *Rewards*: nominal recognition for participating and the reward of learning new knowledge are important in recognizing the participant’s effort, 5) *Reciprocity*: mutual obligation should be negotiated for what is to be provided by recruiters and what is to be expected from participants, 6) *Resolution:* recruitment persistence and a willingness to repeatedly make contact until agreement to participate is eventually reached, and 7) *Respect*: recruiters need to genuinely respect participants, their work, and their constraints. For our research study, several 7R framework strategies were identical for all eligible PCPs. Specifically, eligible PCPs received identical information on anticipated rewards, requirements, and reciprocity expectations.

**Fig. 1:**
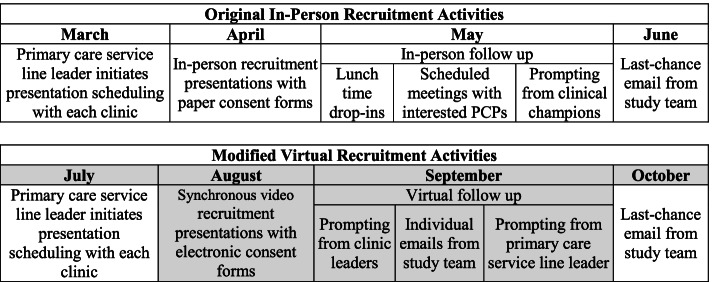
Comparison between original and modified recruitment timeline and activities. *Note*: The gray sections indicate activities that were adjusted when the in-person activities were cancelled. The recruitment period lasted 55 business days

Subsequently, we planned to rely heavily on 1) the research team’s reputation and relationships that stemmed from prior studies to design OneSheet; 2) clinical championship from a PCP research team member, and – crucially – 3) *in-person* presentations and individual interactions with eligible PCPs (see Fig. 1). Following a mass email from the primary care service line leader to eligible PCPs, the study team planned to follow up with in-person recruitment presentations to PCPs and drop-in consultations (e.g., during lunch hours) at each clinic. We then planned to distribute paper-based informed consent forms that eligible PCPs could complete during these interactions. Finally, we planned for a PCP champion, also a member of the research team, to promote the study among colleagues, including announcements and reminders of recruitment during in-person meetings.

### Modified recruitment strategy due to COVID-19 restrictions

Due to the emergent need for physical distancing, the participating health system requested that our study team cancel planned in-person recruitment activities. As originally planned, the primary care service line leader, who is a physician, sent introductory emails connecting the research coordinator to clinic leaders (i.e., clinic manager and chief physician) at each primary care clinic site (see Fig. 1). Next, a research team member with a clinical background gave an 8-min synchronous video presentation at each clinic’s virtual monthly provider meeting. After all presentations occurred, we conducted three iterations of extensive email follow-up. First, the primary care service line leader sent additional emails to each clinic’s leaders asking them to assist in recruiting eligible PCPs at each clinic. Second, each clinic’s leaders contacted eligible PCPs through mass emails, individual emails, or, in rare cases, in-person prompting. Finally, a research coordinator sent a “last chance” email to all eligible PCPs who had not yet consented to participate in the study.

## Measures

To quantify recruitment effectiveness, the research coordinator maintained a detailed spreadsheet of each communication between relevant stakeholders (including the primary care service line leader, clinic leaders, the study team) and eligible PCPs. To minimize measurement bias, the spreadsheet data were entered during our recruitment efforts. From this spreadsheet, we calculated metrics to evaluate the recruitment process, including the number of business days and recruitment contacts needed to enroll a PCP as a study participant. We defined a successful recruitment outcome as receiving a signed informed consent form and a completed demographic questionnaire from an eligible PCP. We also adopted a previously used approach calculating the costs of recruitment [16]. Specifically, we calculated costs based on the role of the primary recruitment personnel (i.e., the study research coordinator, the co-principal investigators, and a co-investigator), percentage of time spent on recruitment, total recruitment hours, and the hourly rate.

## Results

The originally-planned March 2020 start was delayed due to COVID-19 (see Fig. 1). In total, we recruited 45 of 63 eligible PCPs practicing at ten primary care clinic locations. The majority of PCPs were medical or osteopathic physicians (70%), female (73%), white (68%), and not of Hispanic or Latino ethnicity (82%) (see Table 1). On average, recruited PCPs had spent 14 years practicing medicine. The clinical credentials and gender of PCPs who were not successfully enrolled in the study were similar to those recruited.

**Table 1:** Demographic characteristics of primary care providers (PCP) who were recruited as research subjects in an RCT assessing the effectiveness of electronic decision-support tool (*n* = 45)

Characteristic	N, (%)
**Clinical training credentials**	
Medical Doctor or Doctor of Osteopathy (MD/DO)	31 (70)
Physician Assistant (PA)	4 (9)
Advanced Registered Nurse Practitioner (ARNP)	9 (21)
**Sex**	
Female	32 (73)
**Ethnicity**	
Hispanic or Latino	6 (14)
Not Hispanic or Latino	36 (82)
Prefer not to answer	2 (4)
**Race**	
American Indian/Alaska Native	0 (0)
Asian	5 (11)
Native Hawaiian or Other Pacific Islander	0 (0)
Black or African American	5 (11)
White	30 (68)
Prefer not to answer	5 (11)
Years actively practicing medicine (mean, SD)	13.6 (9.3)

Overall, PCP recruitment lasted 55 business days. The synchronous video presentations at the ten clinics’ virtual provider meetings occurred over 31 business days. We spent an average of three business days and sent an average of three emails to clinic leaders before successfully being scheduled to attend a virtual provider meeting (see Table 2). Once the video presentations were completed, the research coordinator exchanged, on average, ten emails with clinic leaders to discuss recruitment-related topics. We also engaged in extensive email follow-up with eligible PCPs. Counting from the date of first contact, which was the video presentation, to the date of consent, we spent on average 17 business days (range: 0–48) and three attempts (range: 1–7) to recruit an eligible PCP. We counted contact attempts as video presentations, emails from the primary care service line leader, clinic leaders, and research team.

**Table 2:** Metrics quantifying primary care provider (PCP) recruitment efforts in an RCT assessing the effectiveness of an electronic decision-support tool

Recruitment Activity	Mean	SD	Min	Max
**Working with clinic leaders (*n*** **= 10 clinics)**				
Business days between requesting presentation time and scheduling with clinic leaders (i.e., managers and chief physicians)	2.8	2.6	0	7
Number of contacts with clinic leaders to schedule presentation	3	1.1	2	5
Business days between a synchronous video presentation and last eligible PCP signing an informed consent form at each clinic	21.9	14.4	0	48
Number of contacts with clinic leaders discussing recruitment	9.4	3.7	2	15
**Reaching eligible PCPs (*n*** **= 63)**				
Business days from first contact with PCP to signed informed consent (*n* = 45 PCPs)	16.51	11.8	0	48
Number of contacts with PCP before signed informed consent (*n* = 45 PCPs)	3.1	1.8	1	7
Number of contacts with non-consented PCPs during recruitment period (*n* = 18 PCPs)	5.33	0.9	4	7

*Note*: Time is measured in business days. Number of contacts includes synchronous video presentations, emails from the study team, emails from the primary care service line leader, clinic leaders, and occasional in-person reminders

In Table 3, we report recruitment rates by recruitment strategy. Prompting from clinic leaders were most effective, leading to 15 successfully recruited PCPs. The researcher-led synchronous video recruitment presentations at provider meetings resulted in 11 recruited PCPs. Research coordinator emails to eligible PCPs shortly after the synchronous video recruitment presentation resulted in seven additional recruited PCPs. Other recruitment strategies collectively, including primary care service line leader emails, led to the remaining 12 recruited PCPs. The research coordinator spent 154 h on the recruitment activities with a total cost of $4004 or $89 per recruited PCP. Additionally, the research coordinator was supported by three team members involved in developing the recruitment materials, attending presentations, and communicating with health system leadership. We estimated that these three team members spent a total of 80 h on recruitment related efforts. Two-thirds of 80 h effort was devoted to recruitment during the preparation period (February–July) and one-third during the recruitment period (August–October). Including the additional effort from these team members, the total recruitment cost was $9092 or $202 per recruited PCP (See Table 4).

**Table 3:** Recruitment Strategies and Resulting Yield of recruited Primary Care Providers (PCPs)(*N* = 45)

Recruitment Strategy	Recruited PCPs^**a**^
Clinic leaders (i.e., managers and chief physicians) email eligible PCPs ^b^	15
Research team member conducts synchronous virtual presentation at already-scheduled virtual PCP meetings	11
Research coordinator sends encouragement emails to eligible PCPs	7
Other^a^	5
Service line leader emails encouragement to eligible PCPs	3
Service line leader emails encouragement to clinic leaders	3
Research coordinator sends “last chance” email	1

*Note*: ^a^A recruited PCP was attributed to a particular research strategy if they completed the informed consent form and a returned a questionnaire within three business days following the use of a recruitment strategy. In rare cases, we were unable to attribute a recruitment to a single strategy

^b^Across ten clinics, clinic leaders reported a total of four in-person contacts with potential PCPs at their location

**Table 4:** Costs associated with PCP recruitment efforts (45 out of 63 eligible PCPs recruited)

Characteristic	Research Coordinator	Co-PI 1	Co-PI 2	Co-I	Sum
Total recruitment time, hours	154	32	32	16	
Hourly rate, average $	$26	$57	$88	$28	
Total recruitment cost	$4004	$1824	$2816	$448	$9092
Average cost per recruited PCP, $	$89	$40.50	$62.50	$10	$202

*Note*: Co-PI-co principal investigator; Co-I: co-investigator. The calculations are based on the salary of each team members and estimated total effort (in hours) dedicated to recruitment activities

Several challenges encountered in recruiting PCPs are described in Table 5. Specifically, given the lack of evidence on how to operationalize the 7R framework via predominantly electronic recruitment, we may not have fully leveraged the *Relationships* and *Reputation* factors to their fullest extent. For instance, research team members could not interact in-person with eligible PCPs nor forge strong relationships with clinic leaders to facilitate recruitment. Furthermore, our electronic recruitment messages, compared to in-person methods, may have diminished *Reciprocity, Resolution,* and *Respect*. For instance, eligible PCPs often attended synchronous video presentations via phone instead of using video conferencing software, limiting their ability to see the slides and the demonstration video. By attending via phone, PCPs may have been more likely to be distracted, engaging in multitasking behaviors, or reluctant to ask clarifying questions.

**Table 5:** The 7R framework of the PCP recruitment in an RCT assessing effectiveness of electronic decision-support tool for patients with chronic pain

R-factors	Electronic recruitment approaches used	Challenges faced
**Relationship**	—Introductory email to clinic leadership by a primary care service line lead. —OneSheet’s demonstration video by clinician champion included in the synchronous video presentation.	—Limited opportunities to form relationships with eligible PCPs, and clinic leadership through frequent in-person check-ins. —Inability to promote the project through in-person, informal chats with eligible PCPs by clinical champion.
**Reputation**	— Synchronous video presentation and electronic consent form articulated appropriate data protection of participant information. —Primary care service line lead and clinician champion emails reinforced team’s reputation and OneSheet’s value.	—Limited options for demonstrating team’s reputation for conducting high quality research.
**Requirements**	— Synchronous video presentations done during provider meetings. —Explanation of the participation burden. —Opportunity to choose preferred communication. —Demonstration of OneSheet’s user-friendliness and clinical effectiveness.	—Uncertainty about PCPs understanding of the reasonable requirements for participating in the study due to low engagement at the synchronous video presentation and lack of responsiveness to follow-up emails.
**Rewards**	—Opportunity to use OneSheet with potential to improve care. —Acknowledgement of PCPs contributions. —Tokens of gratitude for the treatment group	—Limited options for articulating OneSheet’s potential for improving care.
**Reciprocity**	All electronic communication included: —Anticipated burden of participation —Description of support for treatment group —Goal of minimizing workflow disruptions.	—Uncertainty about PCPs understanding of the expectations and team’s support due to low engagement at the synchronous video presentation and lack of responsiveness to follow-up emails.
**Resolution**	—Follow-up emails sent to eligible PCPs by research coordinator, clinic leaders, and a primary care service line lead.	—Lack of responsiveness to follow-up emails. —Overestimating the effectiveness of the synchronous video presentations.
**Respect**	All electronic communication included: —Communication of respect for PCP time and willingness to participate. —Opportunity to stop participation at any point. —Opportunity to choose preferred communication.	—Uncertainty about PCPs receptivity and apprehension of the team’s respect for participating in the study due to lack of responsiveness and low engagement in electronic communication.

*Note*: Expanded definitions of the 7R components: (1) relationship: recruiters need to be known for their involvement in the local medical community and for doing practical research, (2) reputation: recruiters need to be known for doing research. Participants need to believe that the relationship and information will not be abused, (3) requirements: resource demands for participants in study-related activities need to be minimized, (4) rewards: nominal recognition for participating and the reward of learning new knowledge are important in recognizing the participant’s effort, (5) reciprocity: mutual obligation should be negotiated for what is to be provided by recruiters and what is to be expected from participants, (6) resolution: recruitment persistence and a willingness to repeatedly make contact are needed until agreement to participate is eventually reached, and (7) respect: recruiters need to genuinely respect participants, their work, and their constraints. Participation should never be taken for granted

## Discussion

Our study demonstrates that PCPs can be efficiently recruited as *research subjects* at low cost using predominantly electronic approaches, even during the COVID-19 pandemic. Our success can be attributed to the effective modification of recruitment strategies for enrolling healthcare providers described in the 7R framework [5, 11] to electronic modes of recruitment. First, we leveraged existing relationships from prior research projects to connect with key stakeholders. Specifically, a primary care service line leader sent introductory emails to clinic leaders (*Relationship & Reputation*). As with in-person recruitment [5, 16, 20–22], email introductions facilitated connections with each clinic and recruitment activity rollout. However, as with in-person recruitment, successful recruitment required substantial email follow-up with clinic leaders and eligible PCPs. Nevertheless, our recruitment metrics are comparable or better in terms of the number of contacts and days to enroll a PCP relative to previous studies [6, 16]. Thus, despite not having frequent in-person check-ins at the clinics, we were able to establish a good relationship with clinic leaders, which was borne out in our recruitment numbers.

Second, we relied on clinic leaders at each clinic location to assist with recruiting eligible PCPs (*Relationship & Reputation*). As with in-person recruitment [5, 10, 21–23], using such “in-house” clinic leaders, who were either physicians or managers, was an effective strategy in our study. Thus, “in-house” clinic leaders had comparative efficacy in recruiting PCPs even with predominantly electronic modes. Eligible PCPs were more responsive to emails from clinic leaders, resulting in higher recruitment rates relative to emails from the research team. To ease the burden, we shared draft emails to be used by the clinic leaders to approach eligible PCPs. Thus, future studies should explore what specific aspects of electronic communication between clinician champions and eligible PCPs are most effective.

Third, while in-person recruitment methods are generally thought to be most effective [5, 10, 11, 21], we found a number of PCPs enrolled after attending a synchronous video presentation. Our video presentation articulated anticipated requirements, expectations, rewards, and demonstrated respect for PCP’s time (*Requirements*, *Rewards, Reciprocity, and Respect*). Thus, assuming future meetings of eligible healthcare providers are available virtually, video conferencing software holds a strong promise for effectively increasing recruitment efforts. This is notable, especially for studies that span institutions and geographies for which in-person recruitment may be cost prohibitive. However, we faced several unanticipated challenges with recruiting PCPs during synchronous video presentations, including PCPs connecting via phone. This prevented some PCPs from viewing our video presentation and may have reduced their engagement. Given the flexibility, relatively low cost, and effectiveness of the synchronous video presentations, additional research is needed to identify best practices for gaining higher PCP engagement and buy-in during synchronous virtual presentations.

Finally, effective adaptation of the 7R framework allowed us to conduct our recruitment at a relatively low costs, an estimated $9092 or $202 per recruited PCP. Our recruitment costs are substantially lower than one study estimating recruitment costs published to date [16]. Of note, Fagnan et al., reported costs associated with recruiting an entire practice, used multiple recruiters with clinical training and thus higher hourly rates, and had contacted substantially greater number of eligible subjects (*N* = 3669 practices) relative to our study. The cost difference could also be explained by a substantial difference in the scales of the studies. Fagnan’s study enrolled eligible practices from six states across the country relative to only one in our study. Unlike Fagnan’s recruitment costs, our costs calculations also did not include travel time to recruit eligible PCPs, since there were no in-person interactions, potentially contributing to cost-savings. Finally, our research team recruited eligible PCPs from a health system with which the team had an ongoing academic partnership, which may have allowed us to incur lower recruitment costs.

Our study has several strengths. First, to our knowledge, we are the first to report experiences of recruiting PCPs as research *subjects* in an RCT during a pandemic. Second, we demonstrated that recruiting PCPs in an RCT can be done electronically, within a reasonable timeframe, and relatively low cost. Nevertheless, our study is not without limitations. Due to the co-occurring COVID-19 pandemic resulting in high workload and stress among eligible PCPs, we were unable to gather feedback from eligible PCPs on how they perceived our predominantly electronic recruitment strategy. Relatedly, we did not conduct a baseline assessment of several 7R framework strategies, such as PCPs perception of the research team’s reputation for conducting rigorous research, which may have shed more light on the effectiveness of our recruitment efforts. Second, recruited PCPs came from a single health system in a Midwestern state, thus the generalizability of our findings may be limited. Third, we calculated costs per recruited PCPs using only the research coordinator’s salary and effort. We used this approach, consistent with other studies, because of difficulty accurately allocating percent effort of other team members to recruitment. Importantly, the research coordinator’s primary responsibility was recruitment, thus the coordinator’s efforts should adequately capture anticipated costs per recruited PCP. Furthermore, because our recruitment strategies were rolled out sequentially, some strategies may have led to fewer recruited PCPs because fewer eligible PCPs remained at the time of rollout. Finally, as our research team relied heavily on established relationships with primary care leadership and individual clinics, other teams without such connections may experience additional challenges recruiting PCPs electronically.

## Conclusion

PCPs can be efficiently recruited as research subjects using primarily electronic communications, even during time of high workload and stress. Electronic recruitment was shown to be more efficient, both in time and costs. Clinic leader outreach and synchronous video presentations may be particularly useful recruitment strategies.

## Data Availability

The datasets generated and analyzed for the current study are available from the corresponding author on reasonable request.

## Abbreviations

PCP: Primary care provider
RCT: Randomized controlled trial
EHR: Electronic health record
CDC: Centers for Disease Control and Prevention

## References

[1] Wu J, Lewis ET, Barnett PG, Nevedal AL (2020). Instant messaging: an innovative way to recruit primary care providers for qualitative research. J Gen Intern Med.

[2] Asch S, Connor SE, Hamilton EG, Fox SA (2000). Problems in recruiting community-based physicians for health services research. J Gen Intern Med.

[3] Johnston S, Liddy C, Hogg W, Donskov M, Russell G, Gyorfi-Dyke E (2010). Barriers and facilitators to recruitment of physicians and practices for primary care health services research at one Centre. BMC Med Res Methodol.

[4] Sahin D, Yaffe MJ, Sussman T, McCusker J (2014). A mixed studies literature review of family physicians' participation in research. Fam Med.

[5] Riis A, Jensen CE, Maindal HT, Bro F, Jensen MB (2016). Recruitment of general practices: is a standardised approach helpful in the involvement of healthcare professionals in research?. SAGE Open Med.

[6] Hysong SJ, Smitham KB, Knox M, Johnson K-E, SoRelle R, Haidet P (2013). Recruiting clinical personnel as research participants: a framework for assessing feasibility. Implement Sci.

[7] Hummers-Pradier E, Scheidt-Nave C, Martin H, Heinemann S, Kochen MM, Himmel W (2008). Simply no time? Barriers to GPs’ participation in primary health care research. Fam Pract.

[8] Rothwell PM (2005). Subgroup analysis in randomised controlled trials: importance, indications, and interpretation. Lancet..

[9] McDonald AM, Knight RC, Campbell MK (2006). What influences recruitment to randomised controlled trials? A review of trials funded by two UK funding agencies. Trials..

[10] Fulda KG, Hahn KA, Young RA (2011). Recruiting practice-based research network (PBRN) physicians to be research participants: lessons learned from the North Texas (NorTex) needs assessment study. J Am Board Fam Med.

[11] Solberg LI (2006). Recruiting medical groups for research: relationships, reputation, requirements, rewards, reciprocity, resolution, and respect. Implement Sci.

[12] Morgantini LA, Naha U, Wang H (2020). Factors contributing to healthcare professional burnout during the COVID-19 pandemic: a rapid turnaround global survey. PLoS One.

[13] Sharma M, Creutzfeldt CJ, Lewis A (2021). Health-care professionals' perceptions of critical care resource availability and factors associated with mental well-being during coronavirus disease 2019 (COVID-19): results from a US survey. Clin Infect Dis.

[14] Sathian B, Asim M, Banerjee I (2020). Impact of COVID-19 on clinical trials and clinical research: a systematic review. Nepal J Epidemiol.

[15] Broyles LM, Rodriguez KL, Price PA, Bayliss NK, Sevick MA (2011). Overcoming barriers to the recruitment of nurses as participants in health care research. Qual Health Res.

[16] Fagnan LJ, Walunas TL, Parchman ML (2018). Engaging primary care practices in studies of improvement: did you budget enough for practice recruitment?. Ann Fam Med.

[17] Harle CA, Apathy NC, Cook RL (2018). Information needs and requirements for decision support in primary care: an analysis of chronic pain care. AMIA Annu Symp Proc.

[18] Dowell D, Haegerich TM, Chou R. CDC guideline for prescribing opioids for chronic pain—United States, 2016. Jama; 2016;315(15):1624–45. 10.1001/jama.2016.1464 PMC6390846 26977696

[19] Indiana Primary Health Care Association. IPHCA Dashboard for Health Centers in Indiana. 2021. https://www.indianapca.org/resource/resource-link/dashboard-for-health-centers-in-indiana/. Accessed 21 May 2021.

[20] Bower P, Brueton V, Gamble C (2014). Interventions to improve recruitment and retention in clinical trials: a survey and workshop to assess current practice and future priorities. Trials..

[21] Chaudhari N, Ravi R, Gogtay NJ, Thatte UM (2020). Recruitment and retention of the participants in clinical trials: challenges and solutions. Perspect Clin Res.

[22] Goldman V, Dushkin A, Wexler DJ (2019). Effective recruitment for practice-based research: lessons from the REAL HEALTH-diabetes study. Contemp Clin Trials Commun.

[23] Ngune I, Jiwa M, Dadich A, Lotriet J, Sriram D (2012). Effective recruitment strategies in primary care research: a systematic review. Qual Prim Care.

